# Multiple Deep Vein Thromboses After Curative Surgery for Cushing Disease: A Case Presentation and Review

**DOI:** 10.1016/j.aace.2022.08.003

**Published:** 2022-08-05

**Authors:** Marissa N. Contento, Sanah Rana, Erika Brutsaert

**Affiliations:** 1 Department of Endocrinology, New York Medical College, Valhalla, New York; 2 Baycare Medical Group, Clearwater, Florida; 3 Department of Endocrinology, Westchester Medical Center, Hawthorne, New York

**Keywords:** Cushing syndrome, thrombosis, thromboprophylaxis, ACTH, adrenocorticotropic hormone, ATIII, antithrombin III, CD, Cushing disease, CS, Cushing syndrome, F8, factor 8, PAI-1, plasminogen activator inhibitor 1, TAT, thrombin-antithrombin, UFC, urinary free cortisol, vWF, von Willebrand factor

## Abstract

**Background/Objective:**

Cushing syndrome (CS) is a prothrombotic state associated with an increased risk of postoperative venous thrombosis. We aim to present the case of a patient with Cushing disease who underwent pituitary surgery and subsequently developed acute lower extremity deep venous thromboses after anticoagulation was stopped.

**Case Report:**

We present the case of a 57-year-old woman who was admitted for intra-abdominal abscesses after a gastric bypass surgery and was found to have evidence of severe CS. Her 24-hour urinary free cortisol level was 898.6 μg/24 h. She was diagnosed with Cushing disease and underwent transsphenoidal resection of a pituitary adenoma, with an appropriate postoperative drop in the cortisol level. She received thromboprophylaxis during hospitalization; however, this was discontinued upon discharge, on postoperative day 9, because she was ambulating. Five days after hospital discharge and 14 days after her surgery, she developed left lower extremity edema and was found to have 4 deep venous thromboses.

**Discussion:**

As previously described, thrombotic risk can be elevated for at least 1 month after surgery for CS, and thromboprophylaxis can decrease this risk.

**Conclusion:**

This case highlights the need for clear recommendations for the duration of postoperative thromboprophylaxis in patients with CS. Clinicians should consider continuing thromboprophylaxis for at least 1 month after surgery for CS.

**Highlights**

• Postoperative patients with Cushing syndrome (CS) have an increased thrombotic risk
• Thrombotic risk in patients with CS can remain elevated for at least 1 month after surgery
• There are no consistent guidelines for postoperative thromboprophylaxis in patients with CS
• Four postoperative deep venous thromboses occurred in this case 2 weeks after the cortisol level normalized
• Thromboprophylaxis can decrease the risk of thrombosis in postoperative patients with CS

**Clinical Relevance**

This case illustrates that there is an increased risk of thrombosis in patients with Cushing syndrome (CS) after surgery. We review evidence for prolonged thrombosis risk after surgery for CS. This report demonstrates the need for research on risk factors and updated guidelines on postoperative thromboprophylaxis in patients with CS.

## Introduction

Cushing syndrome (CS) is caused by excessive glucocorticoid action. Cushing disease (CD) is a form of CS caused by overproduction of adrenocorticotropic hormone (ACTH) by the pituitary gland. This glucocorticoid excess in patients with CS results in a wide range of pathological changes, including multiple alterations in their coagulation profile.^1^^,^^2^ Previous studies on CS have demonstrated a clear risk of thromboembolic disease; however, the guidelines for prophylaxis are not clear and practice patterns remain variable. Herein, we present the case of a patient with CD who underwent pituitary surgery and subsequently developed 4 acute lower extremity deep venous thromboses (DVTs) after anticoagulation was stopped.

## Case Report

A 57-year-old woman with a medical history of type 2 diabetes and hypertension presented to our institution with progressive abdominal pain 1 month after a gastric bypass surgery for weight loss. The patient had been diagnosed with type 2 diabetes and hypertension 2 years prior to presentation. Abdominal computed tomography showed multiple intra-abdominal abscesses, and therefore, she was started on treatment with intravenous antibiotics and abscess drainage. During hospitalization, she was noted to have hypertension while on her home antihypertensive regimen (160-175 mm Hg/70-95 mm Hg) and hypokalemia, with potassium levels of 1.9 mmol/L, unresponsive to a supplemental potassium chloride level of 60 mEq/d; therefore, the endocrinology service was consulted. On review of systems, she was found to endorse a 2-week history of hirsutism, difficulty climbing stairs, and easy bruising. Her physical examination result was significant for coarse hair on her chin, central adiposity, scattered ecchymoses, and proximal muscle weakness. She had small, supracervical fat pads and a slight dorsocervical hump. No abdominal striae were noted. Further workup revealed a random cortisol level of 45 μg/dL, an ACTH level of 114 pg/mL, and an elevation of urinary free cortisol (UFC) level to 898.6 μg/24 h. After administration of 1 mg of dexamethasone, her cortisol level decreased from 31.6 to 30.6 μg/dL, and after administration of 8 mg of dexamethasone, her cortisol level partially decreased to 11 μg/dL. Brain magnetic resonance imaging result was significant for a pituitary adenoma measuring 6 × 9 × 2 mm^3^. After administration of 1 μg/kg of corticotropin-releasing hormone, the ACTH level rose to 302 pg/mL and the cortisol level rose to 81.5 μg/dL, consistent with a diagnosis of pituitary CD. The patient underwent transsphenoidal resection for the removal of the pituitary tumor, and her postoperative cortisol level was 1.2 μg/dL. Prior to the surgery, the activated partial thromboplastin time (aPTT) was consistently low, ranging from 19.1 to 21.0 seconds, whereas the prothrombin time and international normalized ratio were normal. During hospitalization, she received 5000 mg of unfractionated heparin subcutaneously 3 times daily and was started on twice-daily hydrocortisone. She was able to ambulate and was discharged for home on postoperative day 9; furthermore, heparin was stopped. Her aPTT at the time was 21.8 seconds (low). Five days later, she noted swelling of the left leg and pain and presented to the emergency room, where she was found to have 4 DVTs on the left and was started on apixaban. She underwent a thrombophilia workup 2 months later. A mutational analysis for factor V Leiden and prothrombin factor yielded negative results. The levels of proteins C and S, antithrombin, and antiphospholipid were within normal limits; however, the level of factor VIII (F8) was elevated to 217% (50%-150%). The D-dimer level, which was elevated at the time of thrombosis, had normalized. Six months after her DVTs, her F8 level normalized to 121%.

## Discussion

This is the case of a patient with CD who had an appropriate decrease in the cortisol level and was ambulatory at the time of discharge but developed 4 lower extremity DVTs 14 days after discharge. Hypercoagulability has been clearly described in patients with CS.^3^^,^^4^ After surgery for CS, the risk of venous thromboembolism (VTE) is particularly elevated. The patient in the current case report had also undergone a gastric bypass surgery 1 month prior to presentation; furthermore, the risk of VTE in the general population that has undergone bariatric surgery ranges from 0.2% to 2%, with the highest risk occurring in the first month.^5^ Unlike for orthopedic procedures, there are no clear guidelines for the appropriate treatment for the prevention of VTE in patients with CD. Although some authors recommend prophylaxis for nonambulatory patients only,^6^ many reviews of perioperative management of CS did not address the risk of VTE. Two major guidelines (by the American Association of Clinical Endocrinologists/American College of Endocrinology and the European Society of Endocrinologists/European Network for the Study of Adrenal Tumors) for the management of adrenal incidentaloma also did not address perioperative thromboprophylaxis. Although the 2015 Endocrine Society guidelines recommend that patients with CS be evaluated for risk factors for VTE and that those undergoing surgery receive perioperative prophylaxis for VTE,^7^ these do not provide specific recommendations for risk stratification or the duration of postoperative thromboprophylaxis. However, there may be a shift toward greater recognition of thrombotic risk in patients with CD.^8^^,^^9^ In a recent statement by the Pituitary Society, prophylactic anticoagulation was recommended for individuals with a history of embolism, abnormal coagulation test results, severe hypercortisolism, poor mobility, extended hospital stay, high postoperative cortisol concentrations, or cortisol overreplacement and for those currently using estrogen or oral contraceptive pills.^9^ In addition, in a recent review, Varlamov et al^8^ recommended thromboprophylaxis for 2 to 6 weeks before surgery in patients with CD, with regimens of 2 to 3 months for patients with the highest thrombotic risk. However, the identification of highest-risk patients remains a challenge, and practice patterns still vary significantly among pituitary experts who report prescribing anticoagulation after surgery for anywhere from 1 to 2 days of hospital stay to 2 to 3 months.^9^

The risk of thrombotic events in patients with CS has been reported to be up to 10 to 18 times higher than that in the general population.^4^^,^^10^^,^^11^ This risk appears to increase substantially after surgery,^12^ and in retrospective studies of postoperative patients, VTE occurred in 3% to 20%^1^^,^^10^^,^^13^ of untreated individuals who underwent remission with surgery. The risk of arterial thromboses (myocardial infarction and stroke) is also increased.^14^ Some studies have shown that most complications occur in the first month. Semple and Laws^13^ reported 4 cases of DVT that occurred between 2 and 4 weeks after transsphenoidal surgery for CD, whereas Babic et al^15^ found that VTE occurred in 2.6% of 310 patients who underwent adrenalectomy for CS within 23 days after surgery. It is possible that these studies missed more delayed VTEs given that a retrospective study of 208 patients found that patients with CS have an elevated risk of thromboembolism during 30 to 60 days of the postoperative period.^14^ Several others have also described cases of VTEs occurring 2 to 3 months after curative surgery.^1^^,^^10^

Glucocorticoid excess induces multiple changes in coagulant and thrombotic factors that promote clotting and are likely to be exacerbated during the postoperative state. CS is associated with reduced aPTT; increased activation of several clotting factors, including F8, factor IX, and von Willebrand factor(vWF); increased levels of antithrombin III (ATIII), protein S and C, plasminogen activator inhibitor 1 (PAI-1) antigen and activity, D-dimer, fibrinogen, α(2) antiplasmin, and thrombin-antithrombin complex ; and increased clot lysis time.^1^^,^^4^^,^^16^, ^17^, ^18^ Increases in F8 and vWF levels are predominant changes in patients with CS, explaining the prolongation of aPTT seen in patients with CS. Notably, shorter aPTT has been consistently observed in individuals with CS who develop VTE.^19^

Witek et al^20^ showed that 6 months after successful transsphenoidal management of CD, the levels of both fibrinogen and D-dimer remained elevated despite normalization of cortisol levels. However, in another study comparing preoperative and postoperative coagulative parameters in patients with CS, Manetti et al^18^ found that after successful surgical treatment, the levels of vWF, thrombin-antithrombin complex, ATIII, PAI-1, α(2) and antiplasmin as well as aPTT normalized at 12 months after surgery. In their study, 3 patients (7.5%) who developed VTE had elevated levels of fibrinogen, vWF, PAI-1, and ATIII, with low aPTT, compared with healthy controls. The patient described herein had a shortened aPTT and an increased F8 level, a pattern consistent with thrombophilia caused by hypercortisolism. This abnormality in the F8 level persisted for 2 months but improved by 6 months.

Not surprisingly, studies have shown that a longer duration of thromboprophylaxis after surgery for CS decreases VTE complications. In a retrospective analysis of 307 postoperative patients, among individuals who underwent surgery between 1972 and 1981 without receiving anticoagulants, 15 patients (20%) experienced VTE and 8 (11%) died. In contrast, among individuals who underwent surgery between 1982 and 2000 and received postoperative subcutaneous unfractionated heparin (15 000-22 500 units daily) until clotting parameters normalized, only 6% developed postoperative VTE and only 1 (0.4%) died.^1^ Similarly, Barbot et al^17^ found that there were no postoperative VTEs among 44 patients who received enoxaparin with graduated stockings and early ambulation for 30 days compared with 3 VTEs (among 34 individuals) in those who received only heparin until discharge (or 14 days).

Studies assessing risk factors for VTE have reported inconsistent findings and were limited by small numbers of outcomes and a mix of perioperative and nonperioperative patients. In 1 cohort of 176 patients with active CS, 20 individuals who developed VTE were more likely to have elevated F8 levels, elevated late-night salivary cortisol levels, elevated UFC levels (>10 times the upper limit of normal), shortened aPTT, elevated fibrinogen levels, heterozygosity for factor V Leiden mutation, higher midnight plasma cortisol levels, a previous cardiovascular event, comorbid acute severe infections, reduced mobility, bilateral adrenalectomy, swollen legs and/or varicose veins, obesity, cancer, hypertension, or diabetes mellitus; be older; be men; and be using oral contraceptive pills or hormone replacement therapy and statins.^19^ After a multivariable analysis, the authors created a VTE risk assessment model that assigned 2 points each to age >69 years and reduced mobility and 1 point each to acute severe infection, previous cardiovascular event, a midnight plasma cortisol of >3.15 times the upper limit of normal, and shortened aPTT. A CS VTE score of ≥4 was considered to indicate a very high risk of VTE.^19^ Supporting the notion that thrombotic risk correlates with the degree of hypercortisolemia, several studies have shown a positive association between cortisol and vWF levels.^1^^,^^18^^,^^21^ However, Suarez et al^14^ did not find 24-hour UFC level to be correlated with the development of VTE. In their study, there was also no association between VTE and sex, age, body mass index, smoking, diabetes, hypertension, and estrogen or testosterone.^14^ In a meta-analysis of studies of VTE in patients with CS, there was also no significant association between any thrombotic factors or clinical characteristics, including age, and the development of VTE.^4^

Thrombotic risk persists for at least a month after surgery for CS, and although studies have not consistently identified risk factors for VTE, prolonged thromboprophylaxis has been shown to decrease the risk of VTE. Therefore, it is reasonable to recommend thromboprophylaxis for at least 1 month, especially in cases of severe CS. This case report highlights the need for further research on risk factors associated with thrombosis in patients with CS and the importance of updated guidelines to address this issue.

## Disclosure

The authors have no multiplicity of interest to disclose.

## Consent

The Westchester Medical Center Internal Review Board was informed, and approval was not required for this case report. Patient consent was obtained prior to the submission of the manuscript.
